# Construction, Characterization and Application of Recombinant Porcine Deltacoronavirus Expressing Nanoluciferase

**DOI:** 10.3390/v13101991

**Published:** 2021-10-04

**Authors:** Puxian Fang, Huichang Zhang, He Sun, Gang Wang, Sijin Xia, Jie Ren, Jiansong Zhang, Liyuan Tian, Liurong Fang, Shaobo Xiao

**Affiliations:** 1 State Key Laboratory of Agricultural Microbiology, College of Veterinary Medicine, Huazhong Agricultural University, Wuhan 430070, China; pxfang1990@163.com (P.F.); zhanghuic74@126.com (H.Z.); sunh96@126.com (H.S.); alvin_ssd@webmail.hzau.edu.cn (G.W.); sjx19940618@163.com (S.X.); renjie9507@163.com (J.R.); jszhang545@163.com (J.Z.); 17863802639@163.com (L.T.); fanglr@mail.hzau.edu.cn (L.F.); 2 Key Laboratory of Preventive Veterinary Medicine in Hubei Province, The Cooperative Innovation Center for Sustainable Pig Production, Wuhan 430070, China; 3 Laboratory of Animal Virology, College of Veterinary Medicine, Huazhong Agricultural University, 1 Shi-zi-shan Street, Wuhan 430070, China

**Keywords:** porcine deltacoronavirus, reporter virus, nanoluciferase, antivirals, cell tropism

## Abstract

Porcine deltacoronavirus (PDCoV), an emerging enteropathogenic coronavirus, causes diarrhoea in suckling piglets and has the potential for cross-species transmission. No effective PDCoV vaccines or antiviral drugs are currently available. Here, we successfully generated an infectious clone of PDCoV strain CHN-HN-2014 using a combination of bacterial artificial chromosome (BAC)-based reverse genetics system with a one-step homologous recombination. The recued virus (rCHN-HN-2014) possesses similar growth characteristics to the parental virus in vitro. Based on the established infectious clone and CRISPR/Cas9 technology, a PDCoV reporter virus expressing nanoluciferase (Nluc) was constructed by replacing the NS6 gene. Using two drugs, lycorine and resveratrol, we found that the Nluc reporter virus exhibited high sensibility and easy quantification to rapid antiviral screening. We further used the Nluc reporter virus to test the susceptibility of different cell lines to PDCoV and found that cell lines derived from various host species, including human, swine, cattle and monkey enables PDCoV replication, broadening our understanding of the PDCoV cell tropism range. Taken together, our reporter viruses are available to high throughput screening for antiviral drugs and uncover the infectivity of PDCoV in various cells, which will accelerate our understanding of PDCoV.

## 1. Introduction

Coronavirus (CoVs) infections usually causes respiratory and gastroenteritis illness in humans or animals, and are widely known for their capacity to transmit across species, such as severe acute respiratory syndrome (SARS-CoV), Middle East respiratory syndrome (MERS-CoV) from the human pathogens, and the recently-emerged SARS-CoV-2, and the animal pathogens porcine deltacoronavirus (PDCoV) and swine enteric alphacoronavirus (SeACoV) [1,2,3]. These pathogenic coronaviruses have posed a great threat to public health and animal health. Currently, the family *Coronaviridae* of the order *Nidovirales* has been genetically classified into four genuses, including *Alphacoronavirus*, *Betacoronavirus*, *Gammacoronavirus*, and *Deltacoronavirus*. Among them, PDCoV belongs to the genus *Deltacoronavirus* as a newly emerging porcine enteric coronavirus [4]. PDCoV infection leads to acute diarrhea and often dehydration or even death in nursing piglets, resulting in significant economic losses in pig farm [5,6,7,8].

The PDCoV genome is approximately 25.4 kb in length and encodes a total of 15 mature nonstructural proteins (nsp2-nsp16), four structural proteins (S, E, M, N), and three accessory proteins (NS6, NS7, NS7a) [4,9,10]. The first detection of PDCoV in pig feces was announced in 2012 in Hong Kong [4]. In 2014, its first outbreaks took place in the United states [11,12]. PDCoV was subsequently reported in many states [13] and other countries, such as South Korea [14,15], mainland China [16,17,18], Thailand, Laos, Vietnam [19,20,21], and Japan [22]. PDCoV mainly infects pig populations but is also able to infect chickens [23], turkeys [24] and calves [25]. Recent studies showed that PDCoV could limitedly infect mice in vivo and could be passaged in six-day-old embryonated chicken eggs (ECEs) with high viral replicative efficiency [26,27]. A recent study reported that PDCoV was identified in plasma samples of three Haitian children with acute undifferentiated febrile illness, demonstrating the infection of PDCoV in humans [28]. These studies suggest that PDCoV possesses the potential for cross-species transmissibility. However, to date, the key functional receptor of PDCoV has still not been discovered. Although several studies indicated that porcine aminopeptidase N (p.APN) facilitates the viral entry into non-susceptible cells and promotes viral replication [29,30], APN knockout cells also support PDCoV infection in vitro and in vivo [31,32], suggesting that APN is not a key functional receptor. Many works need to be addressed for an in-depth understanding of the PDCoV infection and its pathogenic mechanism. The reverse genetic system is a powerful technology platform for the exploration of viral infection, pathogenesis and the development of vaccines. Currently, the reverse genetic system from diverse RNA viruses has been successfully constructed and extensively applied through RNA-launched and DNA-launched virus recovery systems [33]. On the basis of reverse genetic systems, many studies have constructed recombinant virus bearing reporter genes, such as Green Fluorescent Protein (GFP), red fluorescent protein [RFP], or luciferase, with extensive advantages, including the analysis of viral characteristics, screening of antiviral drugs, and the detection of the viral host spectrum [34,35,36,37]. Recently, PDCoV infectious cDNA clone and reporter virus expressing GFP have been successfully constructed by RNA-launched and DNA-launched reverse genetic system [38,39]. However, the construction, characterization and application of PDCoV reporter virus expressing other reporter genes with better performance has not been reported until now.

In this study, based on the established infectious cDNA clone derived from the PDCoV CHN-HN-2014 strain, we for the first time developed a PDCoV reporter virus expressing Nluc proteins with a more readily quantifiable and sensitive feature for application to high throughput screening antivirals. In addition, the Nluc reporter virus was applied to detect the infectivity of PDCoV in various cells, demonstrating its broad host tropism. Taken together, it is the first report on the construction, characterization and application of recombinant PDCoV expressing Nluc, which will accelerate our understanding of PDCoV by using it to explore viral infection and pathogenesis mechanisms.

## 2. Materials and Methods

### 2.1. Viruses, Cells and Antibodies

Porcine kidney proximal tubular epithelials (LLC-PK1) were purchased from American Type Culture Collection (ATCC, CL-101) and grown in Dulbecco’s Modified Eagle’s medium (DMEM) (Invitrogen, Carlsbad, CA, USA) containing 10% fetal bovine serum (PAN-biotech, Aidenbach, Germany). Human lung cancer cells (A549, ATCC, CCL-185), African green monkey kidney epithelial cells (Vero, ATCC, CCL-81) and Madin Darby bovine kidney cells (MDBK, ATCC, CCL-22) were cultured in grown in DMEM with 10% fetal bovine serum. PDCoV strain CHN-HN-2014 was isolated from a piglet in China and its GenBank accession number is KT336560 [2]. Alexa Fluor 594-conjugated donkey anti-mouse IgG was purchased from Santa Cruz Biotechnology (Dallas, TX, USA). Mouse monoclonal antibodies (MAbs) against PDCoV N or NS6 have been described in our previous study [9,40].

### 2.2. Assembly of an Infectious cDNA Clone of PDCoV CHN-HN-2014

**Figure 1:**
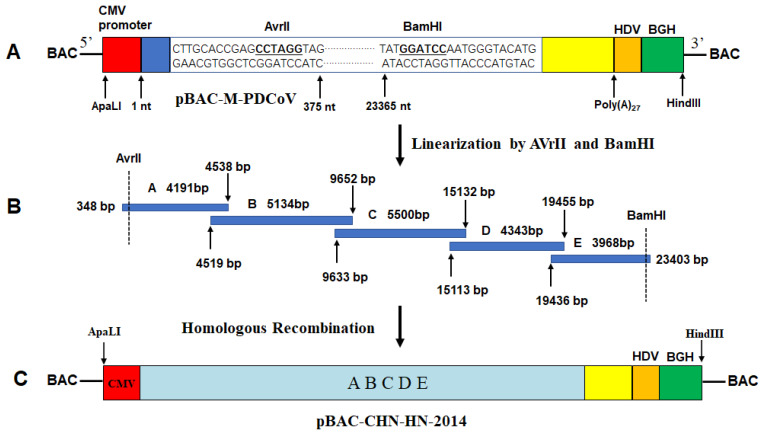
Assembly of a full-length cDNA clone of PDCoV CHN-HN-2014. (**A**) Schematic diagram of the intermediate plasmid pBAC-M-PDCoV. A gene fragment obtained by multi-step fusion PCR, containing CMV promoter, a partial N-terminal region of PDCoV genome (nt 1–375), a partial C-terminal region of PDCoV genome (nt 23,365–25,421), a 27-nt poly(A) tail, the hepatitis deltavirus (HDV) sequence, and the bovine growth hormone (BGH) termination sequences, was cloned into the backbone vector pBeloBAC11 between the restriction sites ApaLI and HindIII. The critical restriction sites and their relative positions in the genome of the PDCoV strain CHN-HN-2014 are indicated. (**B**) Five contiguous genomic overlapping overhangs were amplified and assembled into pBAC-M-PDCoV by homologous recombination. (**C**) Schematic diagram of the full-length cDNA clone of CHN-HN-2014 in the pBeloBAC11 vector, referred to as pBAC-CHN-HN-2014.

Briefly, several vital elements including CMV promoter (initiating viral RNA synthesis), partial N-terminal end of PDCoV genome (nt 1–375, containing a restriction enzyme site of AvrII), C-terminal end of PDCoV genome (nt 23365–25421, containing restriction enzyme site of BamHI), a 27-residue poly (A) tail, hepatitis deltavirus (HDV) ribozyme self-cleavage site and bovine growth hormone (BGH) termination sequences (efficiently stopping the transcription) [41], were cloned into pBeloBAC11 vector through the restriction sites ApaLI and HindIII (Figure 1A), producing an intermediate plasmid (pBAC-M-PDCoV). PDCoV-infected LLC-PK1 cells were subjected to the total RNA extraction using TRIzol reagent (Invitrogen, Carlsbad, CA, USA). Using reverse transcriptase (Invitrogen), RNA was then reverse-transcribed into cDNA via specific primers (A-R, B-R, C-R, D-R and E-R, respectively). The above cDNA were used as templates to amplify five fragments with overlapping 20 nt between each fragment (including A, nt 348–4538; B, nt 4519–9652; C, nt 9633–15132; D, nt 15113–19455; E, nt 19436–23403) with TransStart^®^ FastPfu DNA Polymerase (TransGen Biotech) using primers listed in Table 1 (Figure 1B). In order to distinguish recombinant virus from parental virus, a silent mutation (nt 15121, C→A) was introduced to remove a ClaI site. These PCR products were respectively cloned into pJET1.2/blunt vector (Invitrogen) for obtaining an authentic sequence. Five fragments were ligated into linearized pBAC-M-PDCoV with AvrII and BamHI by homologous recombination using an Infusion Clone Kit (TaKaRa), generating the recombinant plasmid containing PDCoV full-length genome pBAC-CHN-HN-2014 (Figure 1C). The specific primers involved in assembly of pBAC-CHN-HN-2014 are listed in Table 1.

### 2.3. Recovery of Recombinant Virus

LLC-PK1 cells seeded in 6-well plates with 80% confluence were transfected with recombinant plasmids using Lipofectamine^®^ 3000 (Invitrogen) for 4 h. The cells were washed twice and added with DMEM containing 10 μg/mL of trypsin (Sigma), then continually inoculated in a 37 °C, 5% CO_2_ incubator, followed by the daily observation.

### 2.4. Indirect Immunofluorescence Assay (IFA)

Briefly, LLC-PK1 cells seeded in 24-well plates were infected with the indicated viruses. The cells were followed by the harvest and fixation with 4% paraformaldehyde, and permeation with methyl alcohol. Treated cells were processed in an orderly manner according to the following steps, including the blockage of 5% bovine serum albumin and the incubation of primary antibodies and secondary antibodies, respectively. Subsequently, the cells were incubated with 4′,6-diamidino-2-phenylindole (DAPI) for 15 min, followed by the visualization using a confocal laser scanning microscope (Fluoviewver.3.1; Olympus, Tokyo, Japan).

### 2.5. Plaque Assay

LLC-PK1 cells seeded in 6-well plates were incubated with 800 μL of 10-fold serially diluted samples for 1 h at 37 °C, followed by two washes and the addition of 2 mL of DMEM containing 1.5% methylcellulose (15 mg/mL), 10 μg/mL trypsin and 100 U/mL penicillin, 0.1 mg/mL streptomycin. After incubation for the indicated time, the cells were subjected to fixation with 4% paraformaldehyde for 10 min and incubation with 0.1% crystal violet for 5 min.

### 2.6. Viral Growth Curves

LLC-PK1 cells seeded in 24-well plates were infected with the indicated viruses at a multiplicity of infection (MOI) of 0.01 in triplicate wells for 1 h, followed by the washes twice and the addition of fresh DMEM containing 10 μg/mL trypsin. Cell supernatants were harvested at 12, 24, 30, 36, 48 h post infection (hpi) and subjected to TCID_50_ assay [42].

### 2.7. Western Blot Analysis

Infected cells were lysed in 0.1 mL of lysis buffer (promega) for 20 min. Supernatant from lysates were treated and subjected to SDS-PAGE analysis and transmission of proteins to a polyvinylidene fluoride (PVDF) membrane (Millipore, Darmstadt, Germany). The membrane was followed by the blockage of 5% nonfat milk, inoculation with specific primary antibodies (1:2000 dilution for NS6, N and β-actin MAbs), and then horseradish peroxidase (HRP)-conjugated secondary antibodies (1:5000 dilution) (Beyotime, Shanghai, China). Finally, the membrane was visualized by enhanced chemiluminescence reagents (ECR) (Bio-Rad, Hercules, CA, USA).

### 2.8. Quantitative Real-Time RT-PCR

LLC-PK1 cells in 24-well plates were infected with PDCoV for 12 h. Total RNA was extracted from the cells using TRIzol reagent (Invitrogen), followed by first-strand cDNA synthesis using reverse transcriptase. The cDNA was used as the template and subjected to SYBR green PCR assays (Applied Biosystems) with the indicated primers (Table 1) at least three times. The results are expressed as 2^−ΔΔCT^ from quadruplicate samples. Glyceraldehyde-3-phosphate dehydrogenase was used as the reference gene.

### 2.9. Generation of Recombinant Virus Expressing Nluc

To establish a rapid and sensitive anti-PDCoV drug screening platform, a Nluc gene with many preponderant features, including small size, stability, and higher bioluminescence activity was designed to replace the NS6 gene due to its nonessential requirement for normal viral replication [38]. Recombinant PDCoV expressing Nluc was constructed on the basis of CRISPR/Cas9 technology, as described previously [41]. Briefly, two specific primers (sgPDCoV-ΔNS6a and sgPDCoV-ΔNS6b) targeting the upstream (523 bp) and downstream (578 bp) sequences of the NS6 gene were designed and synthesized. Overlapping PCR products amplified respectively with primers of sgPDCoV-ΔNS6a/b and scaffold oligo (Table 2) were used as temples to generate sgRNA-ΔNS6a and sgRNA-ΔNS6b by T7 in vitro transcription. pBAC-CHN-HN-2014 was then cleaved into the linearized BAC vector and a 1.345 kb fragment consisting of NS6 gene, partial sequence of M and N gene according to previous description. At the same time, a fragment (approximately 1.616 kb) containing the Nluc, partial M and N gene sequence, was generated through overlapping PCR using indicated primers (Table 2) and then were respectively ligated into the purified linearized BAC vector via homologous recombination, generating the recombinant BAC plasmid (pBAC-CHN-HN-2014-△NS6-Nluc). Recovery and identification of two recombinant PDCoVs were conducted according to the previous description.

### 2.10. Cell Viability Analysis

Briefly, LLC-PK1 cells were inoculated with different concentrations of lycorine or resveratrol. At 12 h later, cells were subjected to CCK-8–based cell viability assay (Beyotime) according to the manufacturer’s instructions.

### 2.11. Statistical Analysis

GraphPad Prism 5.0 software was used to perform statistical differences analysis using one-way ANOVAs or Student’s t-test. Normal distribution of these was evaluated using the Shapiro-Wilk test. Asterisks indicate the statistical significance. *, *p* < 0.05; **, *p* < 0.01; ***, *p* < 0.001; ****, *p* < 0.0001.

## 3. Results

### 3.1. Recovery and Identification of Recombinant Virus

**Figure 2:**
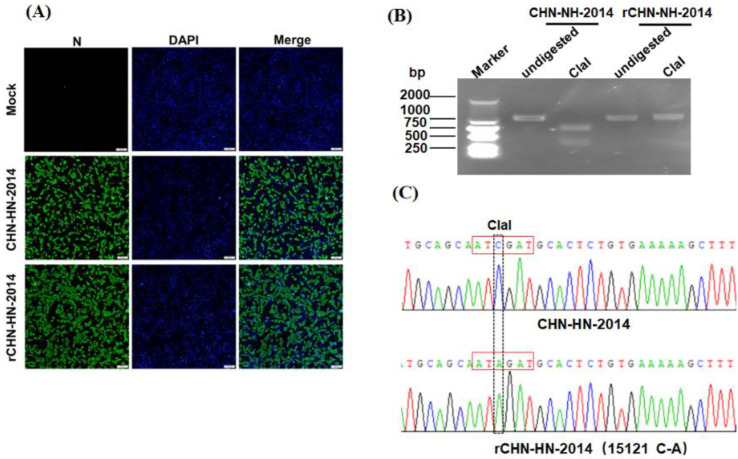
Identification of the rescued PDCoV, rCHN-HN-2014. (**A**) Indirect immunofluorescence analysis of LLC-PK1 cells infected with rCHN-HN-2014 and CHN-HN-2014 at 12 hpi using anti-PDCoV N monoclonal antibody. Bar, 100 μm. (**B**) Differentiation between rCHN-HN-2014 and CHN-HN-2014. The presence of a ClaI restriction site resulted in fragments of 364 bp and 716 bp after digestion with this enzyme. As expected, the restriction site was found in CHN-HN-2014 but not in the rCHN-HN-2014. (**C**) Identification of the introduced molecular marker (15121C→A) in the rCHN-HN-2014 via DNA sequencing. The dotted and red frame represent the molecular marker site and the recognition sequence by ClaI restriction enzymes, respectively.

According to the construction strategy described in Figure 1, a full length cDNA clone of PDCoV strain CHN-HN-2014 was generated successfully. After transfection of LLC-PK1 cells with pBAC-CHN-HN-2014, we observed an obvious cytopathic effect (CPE) that was featured by swelled, rounded, clustered and deciduous cells. After 60 h post-transfection, recombinant virus was harvested and named as rCHN-HN-2014 (designed as ‘passage 1’). IFA assay results displayed specific green fluorescence in rCHN-HN-2014 or parental virus-infected LLC-PK1 cells, but not in uninfected LLC-PK1 cells (Figure 2A). To further verify the identity of rCHN-HN-2014, a genetic marker (C15121 to A15121 mutation) was identified by RT-PCR with the specific primers Clone-F/R (Table 1) and ClaI digestion. As shown in Figure 2B, a 1080-bp fragment (nt 14,761–15,840) consisting of genetic markers from rCHN-HN-2014 could not be digested by ClaI, but homologous fragments from CHN-HN-2014 yielded two fragments of 364 bp and 716 bp by ClaI treatment. In addition, DNA sequencing of the 1080 bp fragment further indicated the presence of genetic markers in the recombinant virus (Figure 2C). Taken together, recombinant virus rCHN-HN-2014 was successfully recovered through the DNA-launched BAC system.

### 3.2. Characterization of Growth Kinetics of CHN-HN-2014 and rCHN-HN-2014

**Figure 3:**
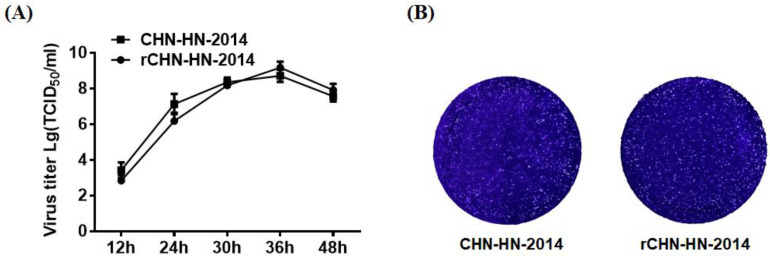
In vitro growth characterization of the CHN-HN-2014 and rCHN-HN-2014. (**A**) Multiple-step growth curves of the CHN-HN-2014 and rCHN-HN-2014 on LLC-PK cells. Cells were infected with CHN-HN-2014 and rCHN-HN-2014 at an MOI of 0.01, and cell supernatants were collected at the various time points post-infection, followed by TCID_50_ assays on LLC-PK cells. (**B**) Plaque morphology and size of the rCHN-HN-2014 in LLC-PK cells are similar to that of the CHN-HN-2014. (Magnification: 10×).

To test whether rCHN-HN-2014 showed similar growth kinetics to those of CHN-HN-2014 in vitro, viral replication was assessed in a multicycle growth experiment. The results indicated that the titers of rCHN-HN-2014 were slightly lower than those of CHN-HN-2014 at 12 and 24 hpi, reaching the highest virus titer at 36 h, after which they declined. At 36 and 48 hpi, the titers of rCHN-HN-2014 were slightly higher than that of CHN-HN-2014 (Figure 3A). The plaque assay results showed that the similar plaques in size and morphology was generated by rCHN-HN-2014 and CHN-HN-2014 (Figure 3B). Taken together, these results strongly indicate that rCHN-HN-2014 and CHN-HN-2014 have similar growth characteristics in vitro.

### 3.3. Recovery of PDCoV Reporter Virus Expressing Nluc

**Figure 4:**
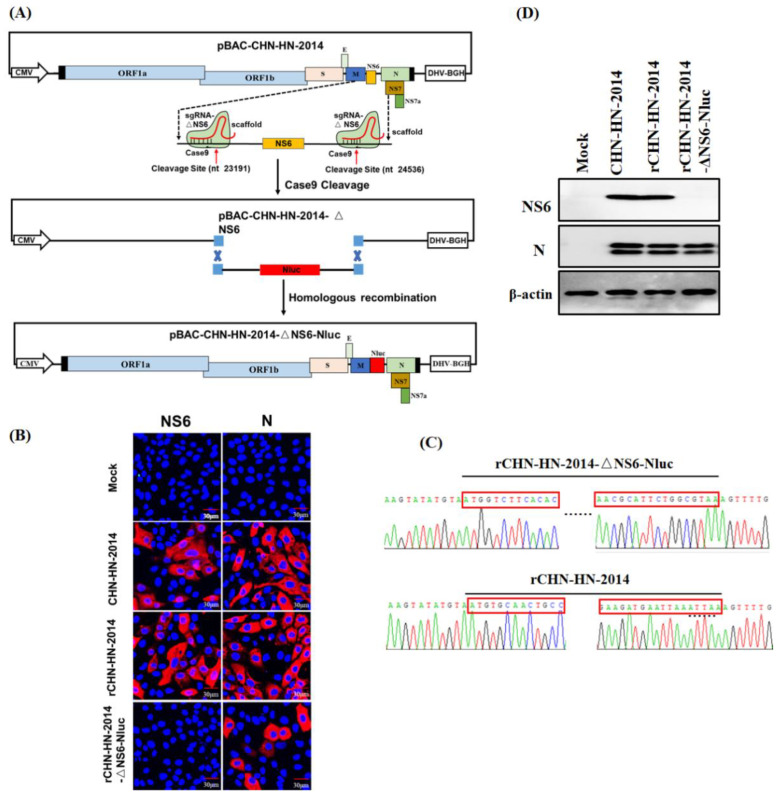
Identification of the rescued PDCoV reporter virus expressing Nluc. (**A**) Schematic diagram of recombinant PDCoV cDNA clone with the replacement of NS6 to Nluc. (**B**) Indirect immunofluorescence analysis of LLC-PK1 cells infected with CHN-HN-2014, rCHN-HN-2014, rCHN-HN-2014-ΔNS6-Nluc at 12 hpi using anti-PDCoV N or NS6 MAbs. (**C**) Mutation identification by sequencing genome of rCHN-HN-2014-ΔNS6-Nluc and rCHN-HN-2014. The red frames represent the partial N-terminal and C-terminal sequences of Nluc or NS6 gene, respectively. (**D**) Western blot assay for lysates from LLC-PK1 cells respectively infected with CHN-HN-2014, rCHN-HN-2014 and rCHN-HN-2014-ΔNS6-Nluc at 12 hpi using anti-PDCoV N and NS6 MAbs. β-actin served as a protein loading control.

Following transfection of the recombinant plasmid pBAC-CHN-HN-2014-△NS6-Nluc (Figure 4A) into LLC-PK1 cells, obvious CPE was observed at 60 h post-transfection. The harvested reporter viruses were designed as rCHN-HN-2014-△NS6-Nluc, followed by the plaque purification. Purified reporter viruses were first subjected to the IFA assay for the infectivity of LLC-PK1 cells using anti-N and NS6 MAbs, respectively. As expected, when IFA assay was performed using anti-N MAbs, PDCoV-specific red fluorescence was observed in cells infected with CHN-HN-2014 and all rescued viruses, suggesting a non-essential role of NS6 for normal viral replication in vitro. When the IFA assay was performed using anti-NS6 MAbs, PDCoV-specific red fluorescence was observed in cells infected with CHN-HN-2014 and rCHN-HN-2014, but not in those infected with Nluc reporter virus (Figure 4B). Furthermore, RT-PCR and DNA sequence analysis of rescued reporter viruses indicated the successful replacement of NS6 gene by Nluc in Nluc reporter virus, respectively (Figure 4C). In accordance with the IFA results, Western blot assay demonstrated that NS6 expression was not observed in Nluc reporter virus-infected cells, and N protein expression was observed in all PDCoVs-infected cells (Figure 4D). Taken together, these results demonstrate the successful recovery of Nluc reporter virus, rCHN-HN-2014-△NS6-Nluc.

### 3.4. Characterization of Growth Kinetics of Nluc Reporter Virus In Vitro

**Figure 5:**
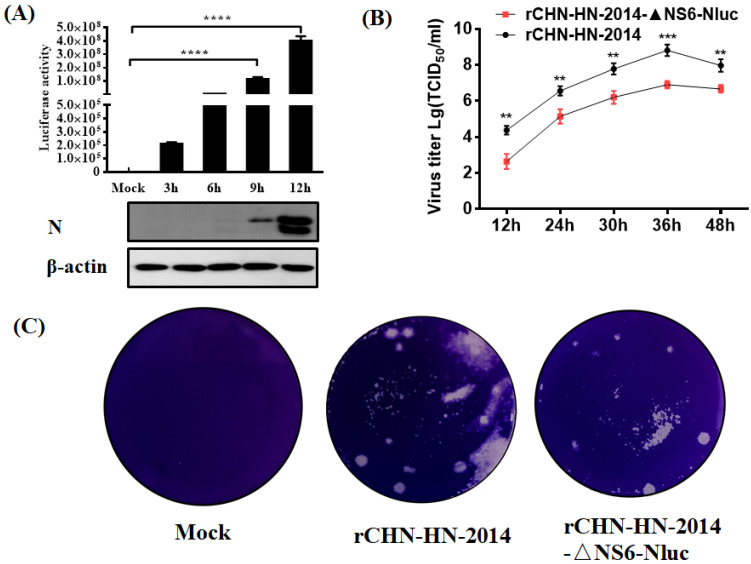
In vitro growth characterization of the Nluc reporter viruses. (**A**) Luciferase activity of rCHN-HN-2014-ΔNS6-Nluc-infected cells. LLC-PK1 cells were infected with rCHN-HN-2014-ΔNS6-Nluc, followed by harvesting infected cells at different time point and measuring luciferase activity. Experiments were carried out in triplicate. Collected cell lysates were subjected to Western blot assay using antibodies against N and β-actin. **** *p* < 0.0001. (**B**) Multiple-step growth curves of the rCHN-HN-2014 and rCHN-HN-2014-ΔNS6-Nluc on LLC-PK cells. Cells were infected with recombinant PDCoVs at an MOI of 0.01, and cell supernatants were collected at the indicated time points post-infection, followed by TCID_50_ assays on LLC-PK cells. ** *p* < 0.01; *** *p* < 0.001. (**C**) Mean plaques size of rCHN-HN-2014-ΔNS6-Nluc in LLC-PK cells are smaller compared with that of rCHN-HN-2014.

To examine the Nluc expression kinetics in vitro, LLC-PK1 cells were inoculated with Nluc reporter virus, and then were harvested to detect the Nluc signals. As shown in Figure 5A, Nluc signals measured at each time point of infection gradually and time-dependently increased, similar to the kinetics of N protein expression in cells infected with Nluc reporter virus. At 3 hpi, an obviously increased luciferase signals were detected compared to the mock-infected control group, although at this time point, the N protein level was barely detected by Western blot. These results showed that the increase in Nluc signals expression was notably correlated with viral proliferation in Nluc reporter virus-infected cells and further demonstrated the high sensitivity of Nluc for reflecting viral replication than N protein expression, especially at the early stage of viral infection. We further examined the growth kinetics of Nluc reporter virus in vitro and found that all recombinant PDCoVs replicated effectively and peaked at 36 hpi. However, lower titers for Nluc reporter virus were observed at all tested time points post-infection compared with that of the rCHN-HN-2014 (Figure 5B). The mean plaques size of Nluc reporter virus in LLC-PK1 cells is smaller compared with that of rCHN-HN-2014 (Figure 5C). These results suggest that Nluc reporter virus possesses the ability to proliferate steadily, and detection of luciferase activity in Nluc reporter virus-infected cells could more sensitively reflect the viral replication levels.

### 3.5. Application of Nluc Reporter VIruses in Drugs Screening

**Figure 6:**
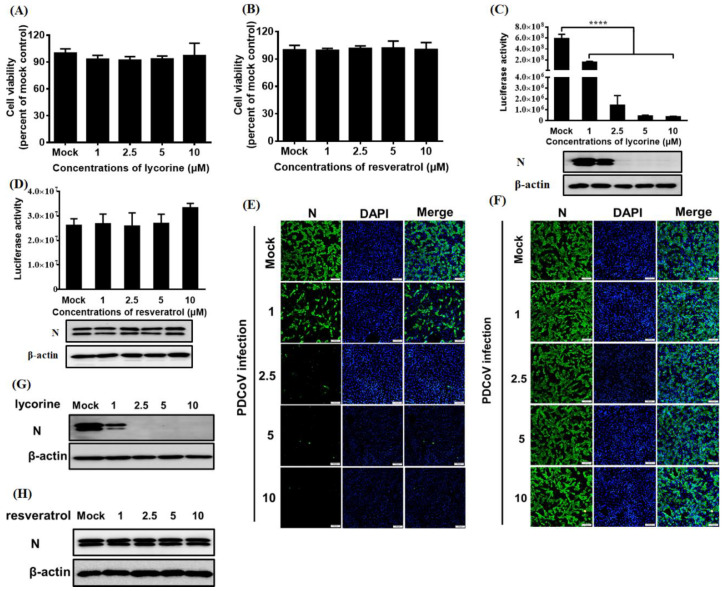
Application of Nluc reporter virus in drugs screening. (**A**,**B**) CCK-8-based cell viability assay for lycorine (**A**) and resveratrol (**B**) as described in materials and methods. (**C**,**D**) The effect of the lycorine (**C**) and resveratrol (**D**) on Nluc reporter virus infection by luciferase activity detection and Western blot analysis. LLC-PK1 cells were pretreated with various concentration of lycorine and resveratrol for 1 h and then infected with Nluc reporter virus for 12 h. Cell lysates were collected and subjected to the measurement of luciferase activity and Western blot assay using antibodies against N and β-actin. **** *p* < 0.0001. (**E**,**F**) The effect of the lycorine (**E**) and resveratrol (**F**) on Nluc reporter virus infection by IFA. LLC-PK1 cells were pretreated with various concentration of lycorine and resveratrol for 1 h and infected with Nluc reporter virus for 12 h, followed by IFA using anti-PDCoV N antibody. Bar, 100 μm. (**G**,**H**) The effect of the lycorine (**G**) and resveratrol (**H**) on wild type PDCoV CHN-HN-2014 infection by Western blot. LLC-PK1 cells were pretreated with various concentration of lycorine and resveratrol as described in panel E and F, and infected with CHN-HN-2014 for 12 h, followed by western blot using anti-PDCoV N and β-actin antibody, respectively.

To investigate the feasibility of the Nluc reporter viruses for antivirals screening, we selected two drugs that were reported to have a negative effect on CoVs replication, namely lycorine [43] and resveratrol [44]. To exclude cytotoxic side effects, cell viability upon lycorine treatment was determined. The result showed that the viability of LLC-PK1 cells remained unchanged with up to 10 μM lycorine (Figure 6A) or resveratrol (Figure 6B). Lycorine treatment significantly inhibited luciferase activity and N protein expression levels in Nluc reporter virus-infected cells in a dose-dependent manner (Figure 6C), but resveratrol treatment had no significantly negative effect on luciferase activity and N protein expression levels in Nluc reporter virus-infected cells (Figure 6D), suggesting a significantly antiviral role of lycorine on PDCoV infection. In agreement with the aforementioned results, IFA results showed that dose-dependent inhibition of viral replication levels was observed in Nluc reporter virus-infected cells with lycorine treatment, rather than in Nluc reporter virus-infected cells with resveratrol treatment (Figure 6E,F). In addition, we further evaluated the effect of lycorine and resveratrol on the wild type PDCoV CHN-HN-2014 strain and demonstrated that the infection of CHN-HN-2014 strain was significantly inhibited by lycorine treatment, but not by resveratrol treatment, in line with these results of their effect on the infection of Nluc reporter virus (Figure 6G,F).These results showed that Nluc reporter virus is suitable for high throughput screening antivirals due to its easily quantifiable feature.

### 3.6. Application of Nluc Reporter Virus in Identifying Permissive Cell Lines

**Figure 7:**
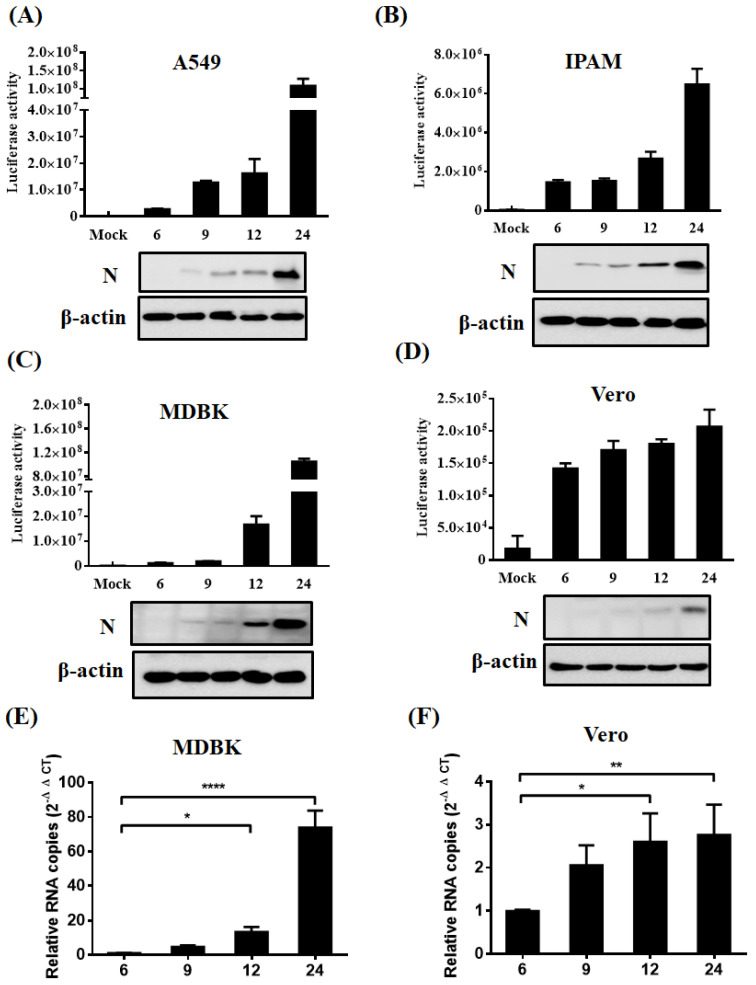
Identification of permissive cell lines. (**A**–**D**) Identification of permissive cell lines via luciferase activity assay and Western blot. A549 (**A**), IPAM (**B**), MDBK (**C**) and Vero (**D**) cells were infected with Nluc reporter virus in the presence of indicated concentration of trypsin. After 6, 9, 12, 24 hpi, cells were harvested and subjected to the measurement of luciferase activity and Western blot assay using antibodies against N and β-actin. (**E**,**F**) RT-qPCR assay for viral infection. MDBK (**E**) and Vero (**F**) cells were infected with Nluc reporter virus as described in panel A, followed by the detection of relative viral mRNA level by RT-qPCR at different time points post infection. * *p* < 0.05; ** *p* < 0.01; **** *p* < 0.0001.

To broaden our understanding of PDCoV cell tropism, we tested the infectivity of PDCoV using Nluc reporter virus in various host cell lines in the presence of trypsin, such as human (A549), swine (IPAM, immortalized cell line of pulmonary alveolar macrophages), cattle (MDBK), and monkey (Vero). Infected cells were collected at various time points (6, 9, 12, 24 hpi) and subjected to the measurement of luciferase activity and N protein expression levels. The results showed that the Nluc reporter virus could infect all tested cells lines derived from various host species (Figure 7A–D). Higher expression of PDCoV N protein was observed in Nluc reporter virus-infected A549, MDBK, and IPAM cells compared to that in Vero at 24 hpi. To further verify the results, we selected MDBK (with the highest viral replication level in the tested four cell lines) and Vero (with the lowest viral replication level in the tested four cell lines) cells to further perform RT-qRCR experiments to detect the viral infection at different time points. In agreement with luciferase activity detection and Western blot results, RT-qPCR also supported the conclusion that PDCoV can infect MDBK and Vero cells evidenced by the significantly increased relative viral mRNA levels in a time-dependent manner (Figure 7E,F). These results indicate that Nluc reporter virus is available to identify the infectivity of PDCoV in various cells and PDCoV has an unusual wide cell tropism, highlighting its potential for cross-species transmission.

## 4. Discussion

Currently, no commercial vaccines and antiviral drugs are able to treat PDCoV infection. Here, we successfully generated a recombinant PDCoV strain rCHN-HN-2014 with similar growth kinetics to that of CHN-HN-2014. We further constructed a PDCoV reporter virus expressing Nluc and demonstrated the availability of Nluc reporter virus with the advantages of its high sensibility and easier quantification to drugs screening and identification of PDCoV susceptible cell lines.

Recent studies have reported the construction of several reverse genetics systems for PDCoV via a T7-based system and a BAC-based system [38,39]. In contrast, combined with a one-step homologous recombination, we used the BAC-based system to more rapidly establish a reverse genetics system for PDCoV. The obtained recombinant virus in our study has a similar growth kinetics and plaque size to that of the wild type virus, which lays an important foundation for further studying of PDCoV (Figure 3). The establishment of reporter viruses play a crucial role in basic research, including studying the process of viral infection, evaluating the efficacy of antiviral drugs, and screening for PDCoV susceptible cell lines. Two types of reporter genes (fluorescent and luciferase genes) have been extensively utilized to produce reporter viruses. Recombinant viruses expressing these reporter genes have been developed to easily study the process of viral infection, to evaluate the efficacy of antiviral compounds, and to perform in vivo studies in animal models. In terms of in vivo imaging applications, luciferase is an attractive reporter because of the inherently low background level in mammalian tissue, but many common luciferases emit blue-shifted light [45]. GFP also displays some limitations due to the absorption of fluorophores’ excitation and emission by hemoglobin and autofluorescence of tissues [36]. However, the reporter gene Nluc used in our reporter virus is the brightest available luciferase, enabling the detection of robust signals despite these limitations [46,47]. Taken together, it is well known that the Nluc protein is more sensitive, easier to quantify, and has other many preponderant features, including its small size, high bioluminescence activity, and ATP independence and stability, making it a kind of reporter protein with better performance than GFP [48,49]. In addition, this is the first report of a PDCoV reporter virus expressing Nluc.

Expression of the reporter gene may serve as an effective surrogate for detecting viral replication in infected cells. Nluc expression from recombinant PDCoV were verified via measurement of Nluc signals (Figure 5A). As expected, Nluc expression showed similar kinetics to viral N protein expression levels, further confirming the potential of reporter genes for functioning as an effective surrogate to evaluate viral replication (Figure 5A). We further demonstrated that the Nluc reporter virus represents a great option for rapidly and simply identifying antivirals just as the significant inhibition of Nluc signals by lycorine treatment, in line with the inhibitory effect of corresponding viral N protein expression levels (Figure 6C). The IFA further supported the significant inhibition of viral replication by lycorine treatment, rather than by resveratrol treatment (Figure 6E,F), displaying the specific inhibitory effect of lycorine on PDCoV infection. These results showed that Nluc reporter viruses are available to high throughput screening for antiviral drugs. Compared to previously reported real-time cell analysis (RTCA) systems for high-throughput screening of antivirals [50], the Nluc reporter virus possesses the obvious advantages with more simpler operation features. A recent study showed that resveratrol inhibits SARS-CoV-2 infection in Vero E6 cells and air–liquid interface cultured human primary bronchial epithelial cells (PBEC) as opposed to human lung epithelial cells (Calu-3), suggesting inherent differences between these different cell line models [44]. However, PDCoV possesses unusual wide cell tropisms. Whether resveratrol displays antiviral activity in other susceptible cell lines needs to be further investigated. Furthermore, our results indicated that Nluc reporter viruses are stable up to six passages in LLC-PK1 cells, as evidenced by RT-PCR for amplification of reporter genes and Western blot for reporter genes expression. Currently, it is the first report to evaluate the feasibility and sensitivity of using Nluc reporter viruses as an antiviral drug screening platform.

Considering that Nluc proteins are readily quantifiable and sensitive, we used the Nluc reporter virus to further investigate PDCoV susceptible cell lines widely used in the laboratory. Except for known susceptible cell lines (ST, IPEC-J2 and IPI-2I) [51,52,53], a total of four cell lines, including human (A549), swine (IPAM), cattle (MDBK), and monkey (Vero), were confirmed to be susceptible to PDCoV (Figure 7). We first provided the experimental evidence that PDCoV can infect A549, IPAM, MDBK cell lines. Combined with luciferase activity values and N protein expression levels, we can speculate that the replication level of PDCoV in Vero cells is lower than in other cell lines (A549, IPAM, MDBK) Interestingly, in addition to reported susceptible human hepatoma (Huh7) and HeLa cells, A549 (lower airway epithelial cell) effectively supports viral replication in the presence of trypsin, implying the potential risk of virus transmission among humans [30,54]. Indeed, recent studies indicated the PDCoV infection in plasma samples of three Haitian children with acute undifferentiated febrile illness [28], but did not definitively identify the zoonotic route of the human infections. More experiments need to be performed for an in-depth understanding of PDCoV infection and transmission among swine population and humans.

In summary, we successfully established the reverse genetic system for the PDCoV strain CHN-HN-2014 and further generated a PDCoV reporter virus expressing Nluc. We confirmed the potential of the Nluc reporter virus to high throughput screening antivirals due to its more readily quantifiable and sensitive features. Furthermore, we identified several experimentally unreported susceptible cell lines to PDCoV infection, expanding the host cell range of PDCoV and confirming the feasibility of the Nluc reporter virus as a powerful tool for identifying susceptible cell lines. Taken together, our successful establishment of a PDCoV reporter virus will contribute to the rapid exploration of viral infection, pathogenesis and the development of effective antiviral drugs.

## Figures and Tables

**Table 1:** Primers used for the RT-PCR amplification of PDCoV full-length genome, the construction of plasmids and real-time RT-PCR assay.

Primer	Nucleotide Sequence (5′-3′)
CMV-F:	GGCGTGCACTTGACATTGATTATTGACTAGTTAT
CMV-R	TAATTTTTATCTTTAGTCCCCATGTACGGTTCACTAAACGAGCTCTGCTTATATAGACC
PDCoV-5′-F	ACATGGGGACTAAAGATAAAAATTATAGCATTAGTCT
PDCoV-5′-R	CTACCTAGGCTCGGTGCAAGG
PDCoV-3′-F	CTTGCACCGAGCCTAGGTAGCTTGCAGGGATTATGGATCCAATGGGTACATG
PDCoV-3′-R	TTGCTCCATCCCCCCTATAAGCCAATTTAATTTCCCC
HDV-F	ATAGGGGGGATGGAGCAAAAAAAAAAAAAAAAAAAAAAAAAAAGGGTCGGCATGGCATC
BGH-R	GAGAAGCTTCCATAGAGCCCACCGCATCCCCAGC
A-F	TAGCACTCCTTGCACCGAGCCTAGGTAGGATAAAACCCCCTAC
A-R	GGCAAATTTAATGGCAGGAC
B-F	GTCCTGCCATTAAATTTGCC
B-R	GTTGTGAATCGATTTGCAAG
C-F	CTTGCAAATCGATTCACAAC
C-R	CAGAGTGCATCTATTGCTGC
D-F	GCAGCAATAGATGCACTCTG
D-R	ACTGCTGGAATTCCTCGTGG
E-F	CCACGAGGAATTCCAGCAGT
E-R	GCACCTCCATGTACCCATTGGATCCATAATCCCTGCAAGGAG
Clone-F	CCAAGTTGGTGATTACATTC
Clone-R	GCTGAGTCAGTGGTCACGCA
qPDCoV-nsp16-F	GCCCTCGGTGGTTCTATCTT
qPDCoV-nsp16-R	TCCTTAGCTTGCCCCAAATA
qGAPDH-F	ACATGGCCTCCAAGGAGTAAGA
qGAPDH-R	GATCGAGTTGGGGCTGTGACT

**Table 2:** Primers used for the construction of recombinant plasmid pBAC-CHN-HN-2014 containing Nluc gene.

Primer	Nucleotide Sequence (5′-3′)
sgPDCoV-△NS6a	TTCTAATACGACTCACTATAGGTCCAAATGGTCACCACTAGTTTTAGAGCTAGA
sgPDCoV-△NS6b	TTCTAATACGACTCACTATAGGTCTTACGACGTACCGCAG GTTTTAGAGCTAGA
scaffold oligo	AAAAGCACCGACTCGGTGCCACTTTTTCAAGTTGATAACGGACTAGCCTTATTTTAACTTGCTATTTCTAGCTCTAAAAC
PDCoV-NS6-upF	GCTCCAACCCTTCACCCTAG
PDCoV-NS6-upR	ATCTTCGAGTGTGAAGACCATTACATATACTTATACAGGCG
Nluc-F	CGCCTGTATAAGTATATGTAATGGTCTTCACACTCGAAGAT
Nluc-R	GATAGATTGGTGTCAAAACTTTACGCCAGAATGCGTTC
PDCoV-NS6-downF	GAACGCATTCTGGCGTAAAGTTTTGACACCAATCTATC
PDCoV-NS6-downR	TTGGGTCTTACGACGTACCG

## Data Availability

The datasets generated for this study are available on request to the corresponding author.
